# Systems toxicology integration uncovers trophoblast apoptosis as a pivotal mechanism underlying PFAS-related recurrent miscarriage

**DOI:** 10.3389/fcell.2026.1889539

**Published:** 2026-07-29

**Authors:** Sisi Wu, Weiwei Dai, Jing Lin, Xunyu Hong

**Affiliations:** Intensive Care Unit, Women and Children’s Hospital of Ningbo University, Ningbo, China

**Keywords:** computational biology, network-based toxicology, PFOA, PFOS, repeated spontaneous abortion, TP53, trophoblast

## Abstract

The present investigation seeks to clarify the molecular underpinnings through which perfluorooctanoic acid (PFOA) and perfluorooctanesulfonic acid (PFOS) precipitate recurrent miscarriage (RM). Initially, prospective targets of PFOA (6050 in total) and PFOS (8896 in total) were systematically compiled from three repositories—CTD, ChEMBL, and Super-PRED—while 1822 pathogenic targets linked to RM were cataloged through the amalgamation of GeneCards, OMIM, and NCBI resources. Venn diagrammatic assessment disclosed 699 overlapping targets shared between PFOA and RM, alongside 832 shared between PFOS and RM. Thereafter, PPI networks were assembled utilizing the STRING platform. By applying median filtering coupled with CytoHubba-based evaluation, central targets were distilled—362 for the PFOA-RM axis and 502 for the PFOS-RM axis—together with their respective top 10 hub genes. Functional enrichment conducted via DAVID pointed toward platelet coagulation elements as potentially critical mediators in PFOA- and PFOS-triggered RM. Moreover, the distinctive enrichment of transcriptional co-regulatory complexes observed specifically for PFOS, coupled with divergent pathway involvements including adipocytokine signaling, TNF cascades, and fluid shear stress transduction between the two agents, implies compound-specific pathogenic modalities. Overlapping target scrutiny pinpointed TP53 as the exclusive pathogenic gene candidate. SMART-based verification affirmed its essential structural domains, with documented subcellular residence in cytoplasmic, nuclear, and mitochondrial compartments. TP53 engages functionally with MDM2 and MDM4 among other interactors, exhibiting pronounced expression within female reproductive tissues such as the cervix and ovary. Notably, its expression proved markedly elevated in healthy villous tissues (encompassing cytotrophoblast and chorionic cavity cells) relative to decidual counterparts. Single-cell interrogation further substantiated prominent TP53 expression within migratory extravillous trophoblasts, extravillous trophoblast populations, and endometrial vascular endothelial cells. *Ex vivo* investigations demonstrated that PFOA and PFOS exposure markedly suppressed HTR8/Svneo cell proliferation while promoting programmed cell death, concomitantly attenuating TP53 protein abundance. Computational molecular docking corroborated favorable binding energetics between both perfluorinated compounds and the TP53 protein. Collectively, these observations furnish novel mechanistic perspectives on PFAS-driven RM pathogenesis, highlighting TP53 as a promising molecular marker for environmentally triggered RM. Also, this study is limited by its *in vitro* nature, the use of a single SV40-immortalized trophoblast cell line, and the absence of primary human trophoblast or organoid validation.

## 1 Introduction

Recurrent miscarriage (RM) is defined as the loss of two or more consecutive pregnancies prior to 20 weeks of gestational age, according to the European Society of Human Reproduction and Embryology (ESHRE) 2022 guidelines. Among women within reproductive age brackets, this condition affects roughly 2.5% of the population. Presently recognized etiological mechanisms encompass chromosomal aberrations, congenital uterine malformations, antiphospholipid syndrome, thrombophilic disorders, endocrine dysregulation, immune system disturbances, infectious agents, among others. Owing to considerable heterogeneity in clinical manifestations along with multifactorial pathogenesis, accurate prediction, definitive diagnosis, and effective therapeutic intervention for RM continue to pose substantial clinical dilemmas. (Qiu et al., 2023; Wei et al., 2022; Li et al., 2023; Wee and Aris, 2023; Bernardini et al., 2021; Fan et al., 2022).

Environmental toxicants have progressively been recognized as significant etiological contributors to RM pathophysiology. Mounting evidence indicates that contact with per- and polyfluoroalkyl substances (PFAS) correlates with elevated incidence of diverse malignancies. A nested case-control investigation drawing from a multi-ethnic American cohort revealed that elevated PFOS exposure was linked to heightened susceptibility to gastrointestinal neoplasms. PFAS constitute a broad category of manufactured compounds extensively utilized across industrial applications and consumer merchandise, distinguished by pronounced environmental persistence and pronounced bioaccumulative capacity. Their biological half-lives extend from multiple years to several decades, and their propensity to concentrate within human tissues—particularly hepatic and reproductive organs—carries profound health implications. (Criswell et al., 2024; Liu et al., 2024; Wijayahena et al., 2025).

From a mechanistic standpoint, PFAS intoxication perturbs metabolic equilibrium within essential human organs including the liver, triggers oxidative cellular stress responses, and alters critical signal transduction cascades, consequently fostering neoplastic transformation. As an illustration, experimental investigations have demonstrated that PFAS can stimulate tumor-promoting signaling routes (for instance, PI3K-Akt) and compromise the activity of tumor surveillance mechanisms, thereby accelerating malignant progression. (Su et al., 2025; Zhou et al., 2025).

It is noteworthy that the bulk of prior investigations concerning PFAS and pregnancy outcomes has concentrated predominantly on gestational complications and hereditary analyses, whereas the molecular basis underlying PFAS-provoked RM remains substantially uncharacterized. A substantial body of literature has validated the reproductive and developmental hazards posed by PFAS. Population-based studies imply that PFAS contact can engender adverse reproductive health consequences among individuals of childbearing potential, encompassing diminished fecundity, pregnancy-related morbidities, and unfavorable neonatal outcomes (including preterm delivery and reduced birth weight). Recent prospective cohort studies published in 2024–2025 further strengthen these associations, reporting dose-response relationships between maternal PFAS burden and early pregnancy loss (Wikström et al., 2024; Joha et al., 2025). In pregnant populations, median serum concentrations of PFOA and PFOS typically range from 1–3 ng/mL and 3–8 ng/mL, respectively, based on recent prospective cohort data, underscoring the real-world relevance of laboratory exposure models.

Structurally, PFOA possesses a carboxylate head group and a linear perfluorinated octyl chain (C8), whereas PFOS bears a sulfonate head group attached to the same C8 perfluorinated backbone. These head-group differences confer distinct electrostatic profiles and protein-binding propensities: the carboxylate moiety of PFOA behaves as a weaker hydrogen-bond acceptor yet exhibits stronger metal-chelation capacity, while the sulfonate head of PFOS is more strongly acidic and can engage in robust electrostatic and halogen-bonding interactions. Consequently, PFOA and PFOS may display divergent target spectra and pathway profiles despite their shared fluorinated tail, a premise that the present network analysis explicitly evaluates.

**FIGURE 1:**
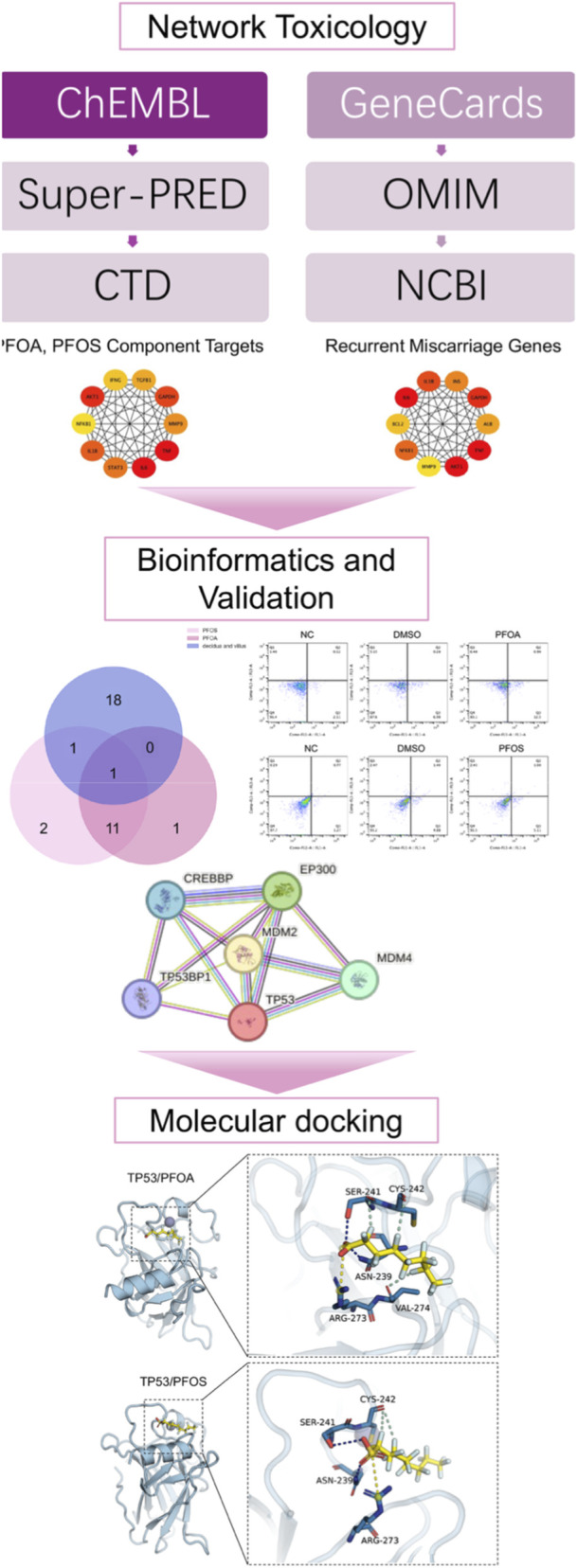
Schematic workflow of the network toxicology investigation of PFAS in recurrent miscarriage.

In light of the distinctive pathological hallmarks characterizing RM and the escalating worldwide impact of PFAS contamination, deciphering the modulatory influence of PFAS on RM initiation and progression assumes paramount importance. Recent studies have demonstrated that multi-omics integration and single-cell resolution profiling are powerful strategies for dissecting complex pathological mechanisms and identifying actionable therapeutic targets across diverse malignancies (Xu et al., 2026; Zhao et al., 2026; Asim, 2026). Inspired by these advances, the current investigation employs an integrated analytical framework encompassing network-based toxicology, cellular experimentation, and molecular docking simulations to probe the molecular basis of PFAS-elicited RM development. We hypothesize that PFOA and PFOS directly bind to the TP53 protein, destabilize its expression or stability, and thereby disrupt apoptosis control in trophoblasts, ultimately contributing to RM pathogenesis. Through this multi-pronged investigative approach, our objective is to elucidate the reproductive hazards attributable to PFAS and its structural analogs, with the ultimate aim of informing preventive and therapeutic modalities for RM management. To comprehensively investigate the molecular connections linking PFAS (specifically PFOA and PFOS) exposure with RM pathogenesis, we devised a multi-phase analytical pipeline for this investigation (depicted in Figure 1).

## 2 Materials and methods

### 2.1 Compilation of PFOA and PFOS target repositories

The Comparative Toxicogenomics Database (CTD) furnishes experimentally substantiated toxicological relationships between PFAS agents and their molecular targets; ChEMBL delivers quantitative interaction metrics, whereas Super-PRED augments coverage by incorporating previously unreported targets, thereby guaranteeing toxicological precision while circumventing redundancy inherent in platforms such as STITCH or SwissTargetPrediction. This procedure entailed extraction of candidate targets susceptible to PFOA and PFOS perturbation from the CTD, ChEMBL, and Super-PRED repositories. (Davis et al., 2021; Zdrazil et al., 2024; Gallo et al., 2022).

### 2.2 Assembly of RM-Associated target datasets

The disease-related target compendium was principally assembled through GeneCards, OMIM, and NCBI repositories. Queries were executed across these platforms employing the search term “RM”. To guarantee robust relevance of retrieved genes to the pathological condition, the relevance scoring threshold within GeneCards was configured to preserve the uppermost 2000 entries. Targets originating from these three sources were consolidated and redundant entries eliminated to generate a disease-specific target inventory for RM.

### 2.3 Determination of overlapping targets among PFOA, PFOS, and RM

A web-based Venn diagram generation utility was employed to delineate overlapping targets between compound-specific and disease-associated target sets. The PFOA, PFOS, and RM target compilations were uploaded to the platform, and resultant intersections were acquired following submission processing (Tang et al., 2023).

### 2.4 Construction of protein-protein interaction (PPI) networks

The intersection targets derived from Venn analysis were subsequently fed into the STRING database for PPI network assembly. The organism parameter was designated as *Homo sapiens*, and network diagrams were produced applying an interaction confidence threshold of ≥0.4. STRING-derived outputs were imported into Cytoscape for network depiction and analytical evaluation, facilitating the computation of topological parameters and generation of PPI network visualizations. Central targets mediating PFOA- and PFOS-induced RM were selected based on nodes demonstrating betweenness centrality surpassing the median value, stress values exceeding the median, and degree connectivity above the median. The CytoHubba plugin was deployed to forecast the top 10 hub genes through the betweenness centrality algorithm. (Szklarczyk et al., 2019; Shannon et al., 2003)

### 2.5 Gene ontology and KEGG pathway enrichment analyses

The DAVID platform was utilized to execute GO (encompassing Biological Process, Molecular Function, and Cellular Component categories) together with KEGG pathway enrichment analyses on the central targets, aiming to identify signaling routes implicated in PFOA- and PFOS-mediated RM pathogenesis. This analytical workflow sought to characterize and emphasize pivotal transduction pathways within the biological processes governing PFOA- and PFOS-provoked RM. Graphical representations were generated to facilitate effective interpretation and presentation of GO and KEGG analytical findings, with charts constructed through online bioinformatics visualization utilities. This methodological approach specifically explored divergent pathways among overlapping targets shared by PFOA, PFOS, and RM grounded in enrichment outputs (Huang et al., 2009).

### 2.6 Prioritization of critical target genes

Following enrichment analysis and hub gene identification procedures conducted in Cytoscape, a subsequent refinement of central target genes was undertaken by computing intersecting elements through Venn diagram methodology.

### 2.7 The Human Protein Atlas resource

The Human Protein Atlas serves as an integrated transcriptomic, proteomic, and systems biology repository capable of mapping protein distribution across diverse tissues, organs, and cellular populations. It harbors protein expression datasets for both neoplastic and normal tissue specimens. In this investigation, hematoxylin-eosin staining evaluation, protein expression quantification, and single-cell transcriptomic analysis of genes under investigation were performed utilizing this resource. All HPA images were retrieved from https://www.proteinatlas.org (accessed on 15 January 2025) and are reproduced under the Creative Commons Attribution-ShareAlike 3.0 International License. The specific HPA antibody identifiers for TP53 are HPA001899 (cervix), HPA001899 (ovary), CAB016046 (placenta), and CAB016046 (endometrium). (Colwil et al., 2011).

### 2.8 Cell culture procedures

The human trophoblastic HTR8/SVneo cell line was propagated in RPMI 1640 growth medium enriched with 10% fetal bovine serum (FBS; Gibco, Grand Island, United States) within our laboratory facility. This cell line was procured from ATCC and authenticated through short tandem repeat (STR) profiling, with results matching the ATCC reference profile. HTR8/SVneo is an SV40-large-T-antigen-immortalized first-trimester extravillous trophoblast line. Because SV40 large T antigen can interfere with p53 signaling, we acknowledge that this model may exhibit altered baseline p53 pathway activity; nevertheless, it remains a well-established and widely accepted model for studying trophoblast biology and environmental toxicant responses. All cultures were sustained under standard incubation parameters (37 °C, 5% CO_2_ atmosphere) and were subcultured for fewer than 60 days post-resuscitation.

### 2.9 Antibodies and chemical reagents

The primary antibodies employed in this study included: TP53 (catalog no. DF7238) and GAPDH (catalog no. AF7021) (both from Affinity, China). PFOA and PFOS standards were procured from Sigma-Aldrich (Shanghai, China).

### 2.10 MTT cell viability assessment

HTR8/SVneo cells were plated onto 96-well culture plates at a density of 1 × 10^4^ cells per well. Triplicate wells were established for each experimental condition. Culture medium was refreshed on alternate days. Following 24 h of compound exposure, metabolically active cells were quantified via the MTT colorimetric assay. In brief, cells were incubated with 50 µL of 0.2% MTT reagent for 30 min at 37 °C within a humidified 5% CO_2_ incubator. Optical density at 560 nm was measured employing a microplate spectrophotometer (Dynex Technologies), and acquired data were subjected to one-way analysis of variance (ANOVA) with Tukey’s post hoc test. PFOA and PFOS were dissolved in dimethyl sulfoxide (DMSO) to prepare stock solutions. The final DMSO concentration in all treatment groups, including vehicle controls, did not exceed 0.1% (v/v), a level previously verified to exert no measurable cytotoxicity in this cell line. Exposure concentrations ranged from 10 to 100 μM, selected to bracket environmentally relevant serum levels (ng/mL range) and prior literature IC_50_ values, thereby enabling mechanistic exploration while acknowledging that upper concentrations exceed typical human exposure.

### 2.11 Flow cytometric analysis

HTR8/SVneo cells were grown for a 24 h period. Upon completion of 24-h drug treatment, collected cells were rinsed with phosphate-buffered saline (PBS) and preserved in ice-cold 70% ethanol at 4 °C overnight. Preserved cells were reconstituted in PBS supplemented with 0.2 mg/mL RNase and 10 µM propidium iodide (PI), then incubated under light-protected conditions for 30 min at 37 °C. A FACSCalibur flow cytometer (Becton Dickinson, San Jose, CA) was employed for flow cytometric acquisition, and resulting data were analyzed through one-way ANOVA with Tukey’s test.

### 2.12 RT-qPCR and immunoblotting procedures

Transcript abundance was determined by quantitative PCR following the manufacturer’s supplied protocols. Immunoblot analysis was similarly executed according to the manufacturer’s specifications. Post-collection, cellular material was lysed in extraction buffer on ice, fortified with phenylmethylsulfonyl fluoride, phosphatase inhibitor cocktail, and protease inhibitor blend. Immunoblot procedures were conducted as detailed in the instruction manual. Chemiluminescence-based detection was performed using a Tanon 5,200 imaging system (Tanon, China). For quantitative assessment of target protein expression, band intensities corresponding to the protein of interest and loading control were quantified using image analysis software, and normalized ratios were computed and evaluated through one-way ANOVA with Tukey’s test.

### 2.13 Molecular docking simulation of compound-target binding

Three-dimensional structural coordinates for PFOA and PFOS were retrieved from the PubChem repository (https://pubchem.ncbi.nlm.nih.gov/). Structural format conversion was accomplished using OpenBabel software. Molecular docking and molecular dynamics simulations have been extensively validated as robust computational approaches for predicting ligand-protein interaction patterns and refining binding hypotheses (Yang et al., 2018; Du et al., 2020). Docking simulations were executed with AutoDock Tools version 1.5.7. Docking pose visualization and analysis were carried out using the Protein-Ligand Interaction Profiler. The acceptance threshold for docking poses was established at a binding energy of < −4.25 kcal/mol (Eberhardt et al., 2021; Liu et al., 2023; Ravindranath et al., 2015; Lee et al., 2020; Song and Yang, 2025).

Molecular dynamics (MD) simulations were performed using AMBER20 to corroborate docking outcomes. Each ligand-protein complex was solvated in an explicit TIP3P water box with a 10-Å buffer, neutralized by counterions, and subjected to energy minimization followed by equilibration. Triplicate production runs of 100 ns each were executed with a 2-fs time step under periodic boundary conditions in the NPT ensemble (300 K, 1 bar). Convergence was assessed by monitoring root-mean-square deviation (RMSD) plateau, radius of gyration (Rg) stability, and hydrogen-bond occupancy across all three independent trajectories.

### 2.14 Ethical considerations

Ethical approval and informed consent: This investigation was performed in strict accordance with the ethical principles outlined in the Declaration of Helsinki. Bioinformatics analyses received ethical clearance from the Institutional Review Board of Ningbo University Affiliated Women and Children’s Hospital.

### 2.15 Statistical methodology

All quantitative data are expressed as arithmetic means ± standard deviation (SD) derived from a minimum of three independent biological replicates. Pairwise group comparisons were performed using Student’s t-test, whereas multi-group comparisons were conducted via one-way ANOVA with Tukey’s post hoc test. Bivariate associations were evaluated using Spearman’s rank correlation coefficient. All statistical computations were executed with SPSS (version 20, IBM Corp., Armonk, NY, United StatesA) and GraphPad Prism 8.0 software packages. Statistical significance thresholds were defined as follows: *p < 0.05, **p < 0.01, ***p < 0.001, and ****p < 0.0001.

## 3 Results

### 3.1 Target compilation for PFOA and PFOS

We initially conducted comprehensive queries within the CTD repository for all documented targets of “PFOA”, yielding 8803 targets, supplemented by 3,215 targets from ChEMBL and 125 targets from Super-PRED. Following deduplication procedures, a consolidated PFOA target repertoire comprising 6050 unique entities was established. Analogously, 15,256“PFOS” targets were recovered from CTD, 1,056 from ChEMBL, and 150 from Super-PRED, culminating in 8896 non-redundant PFOS targets after duplicate elimination.

### 3.2 Curation of recurrent miscarriage pathogenic targets

The following repositories were consulted to assemble recurrent miscarriage pathogenic targets: GeneCards yielded 1,644 targets, OMIM contributed 389 targets, and the NCBI database returned 783 targets. Consolidation of these three sources followed by duplicate removal produced a non-redundant set of 1822 targets associated with recurrent miscarriage.

### 3.3 Overlapping target assessment of PFOA, PFOS, and RM

**FIGURE 2:**
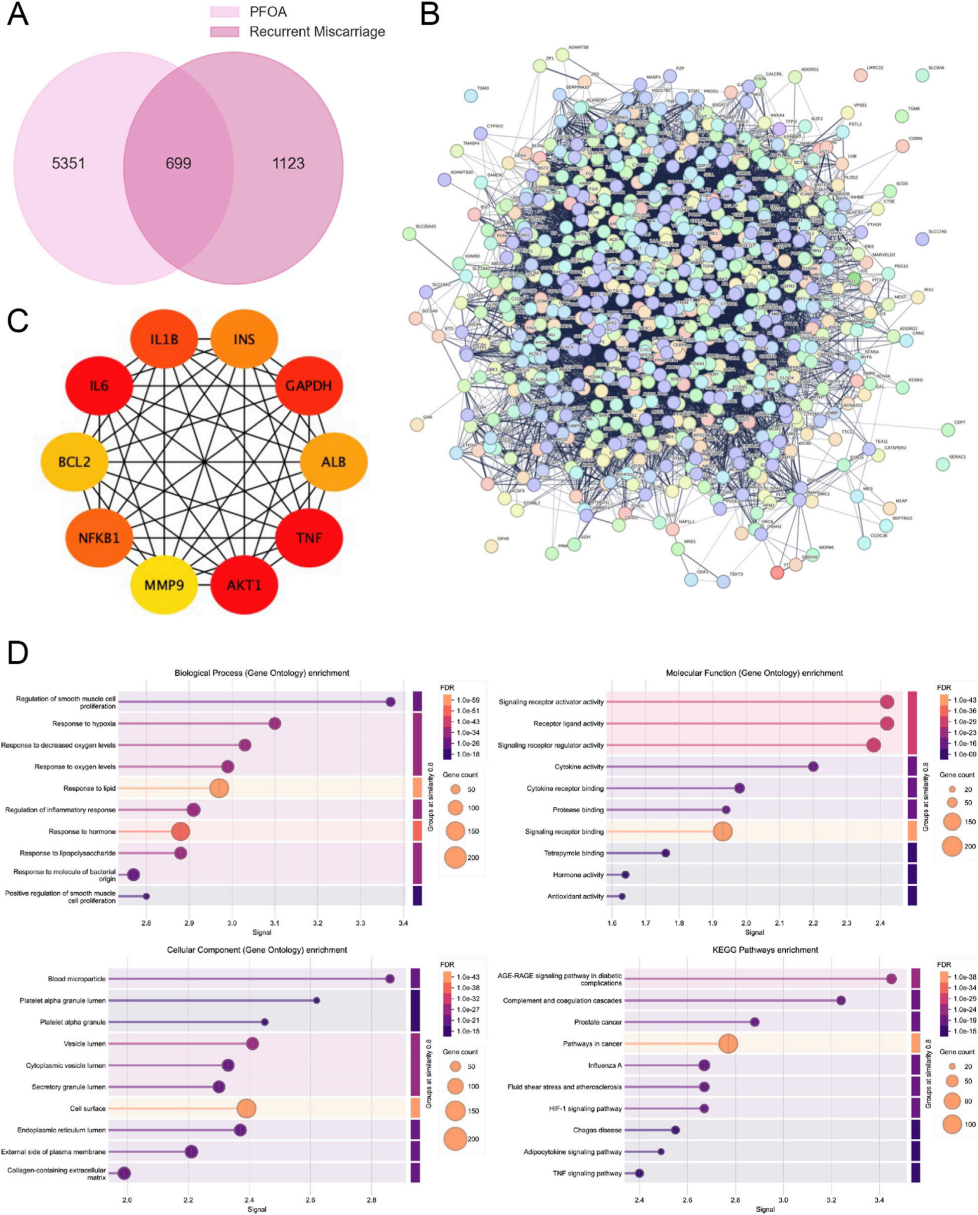
Network toxicology assessment of PFOA-mediated toxicological mechanisms in RM. **(A)** Venn diagram illustrating the overlap between PFOA-associated targets and RM-associated targets. **(B)** Protein-protein interaction map following network filtering. **(C)** Protein-protein interaction network depicting the top 10 hub genes. **(D)** BP-CC-MF and KEGG enrichment analysis.

**FIGURE 3:**
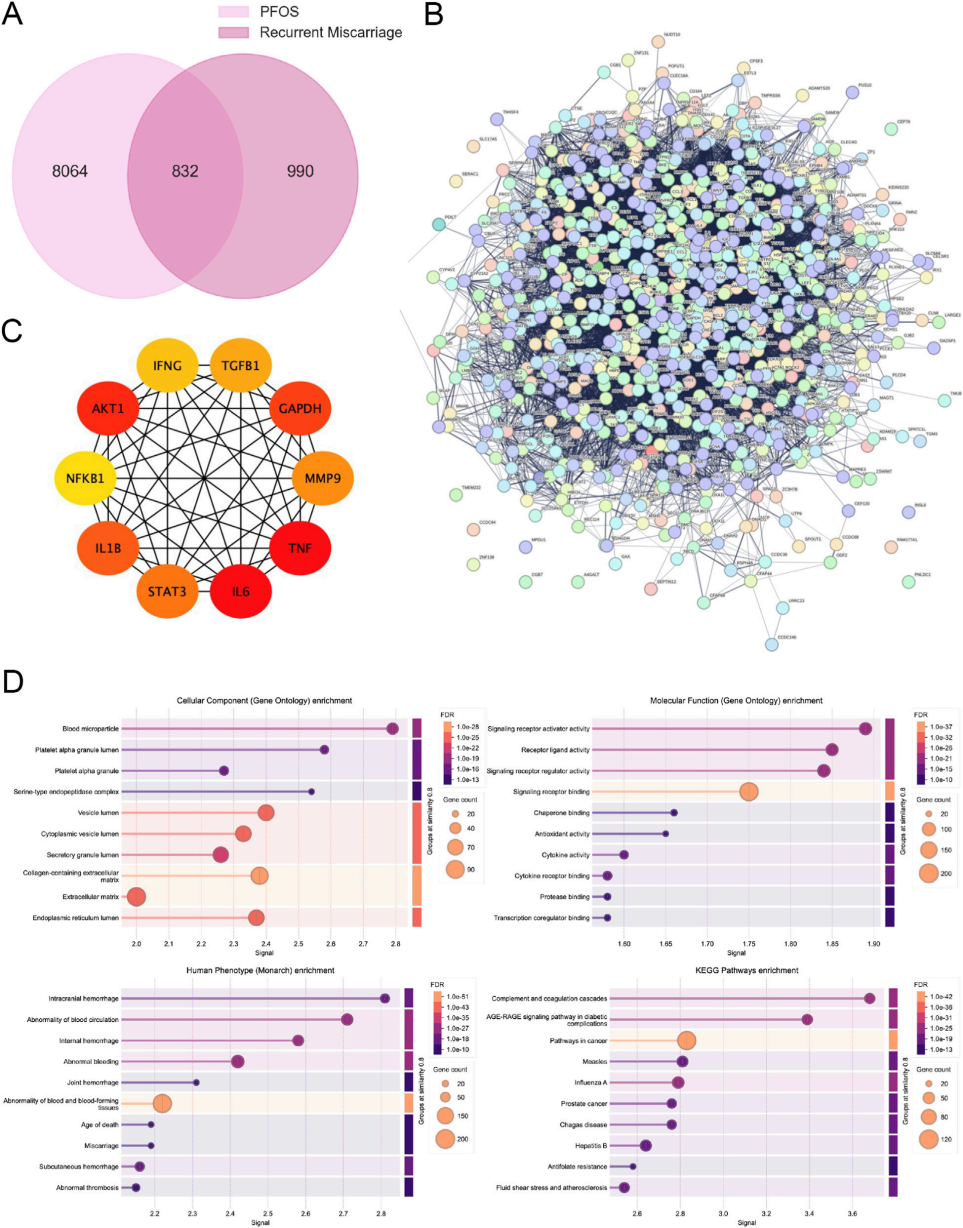
Network toxicology assessment of PFOS-mediated toxicological mechanisms in RM. **(A)** Venn diagram illustrating the overlap between PFOS-associated targets and RM-associated targets. **(B)** Protein-protein interaction map following network filtering. **(C)** Protein-protein interaction network depicting the top 10 hub genes. **(D)** BP-CC-MF and KEGG enrichment analysis.

Venn diagrams were generated to visualize intersecting targets between PFOA and RM (Figure 2A) and between PFOS and RM (Figure 3A). The analysis revealed 699 shared targets at the intersection of PFOA and RM, and 832 shared targets at the intersection of PFOS and RM.

### 3.4 PPI network analysis

Subsequent to data compilation, the STRING database was leveraged to construct PPI networks for the intersecting targets following median-based filtering. Post-filtering analysis identified 362 central targets at the PFOA-RM intersection (Figure 2B), while 502 central targets were identified for the PFOS-RM intersection after analogous median filtering (Figure 3B). Additionally, CytoHubba-based computation disclosed the top 10 hub targets ranked by betweenness centrality (Figures 2C, 3C).

### 3.5 Gene ontology and KEGG pathway enrichment analyses

To achieve more profound insight into the central pathogenic targets, functional enrichment analyses encompassing GO and KEGG pathways were executed on the 362 and 502 core targets respectively via the DAVID platform. The ten entries exhibiting the lowest P-values, together with their associated hit counts and enrichment scores, were selected for graphical representation as bar charts in the GO enrichment analysis (Figures 2D, 3D). Core targets responsive to PFOA and PFOS were prominently enriched within fundamental biological processes including cellular proliferation and platelet-derived microparticle aggregation. Platelet coagulation constituents may represent critical mediators in the onset of PFOA- and PFOS-associated RM. Signal receptor activation events and receptor ligand activities likely constitute the principal molecular functions through which PFOA and PFOS participate in RM development. Of particular note, among PFOS-related molecular functions, transcription co-regulatory complexes emerged as uniquely associated with PFOS, implying a distinct transcriptional regulatory paradigm in PFOS-induced RM pathogenesis.

Within KEGG pathway analysis, numerous cancer-related pathways demonstrated prominent enrichment, suggesting that PFOA and PFOS may operate through neoplastic mechanisms as one facet of their pathological activity (Figures 2D, 3D). Concurrently, the AGE-RAGE signaling axis and complement cascade also exhibited significant enrichment. However, pathway associations specific to PFOA targets showed enrichment in adipocytokine signaling and TNF pathways that diverged from PFOS-associated enrichment in fluid shear stress signaling and folate drug resistance pathways.

### 3.6 Investigation of the pathogenic target TP53

**FIGURE 4:**
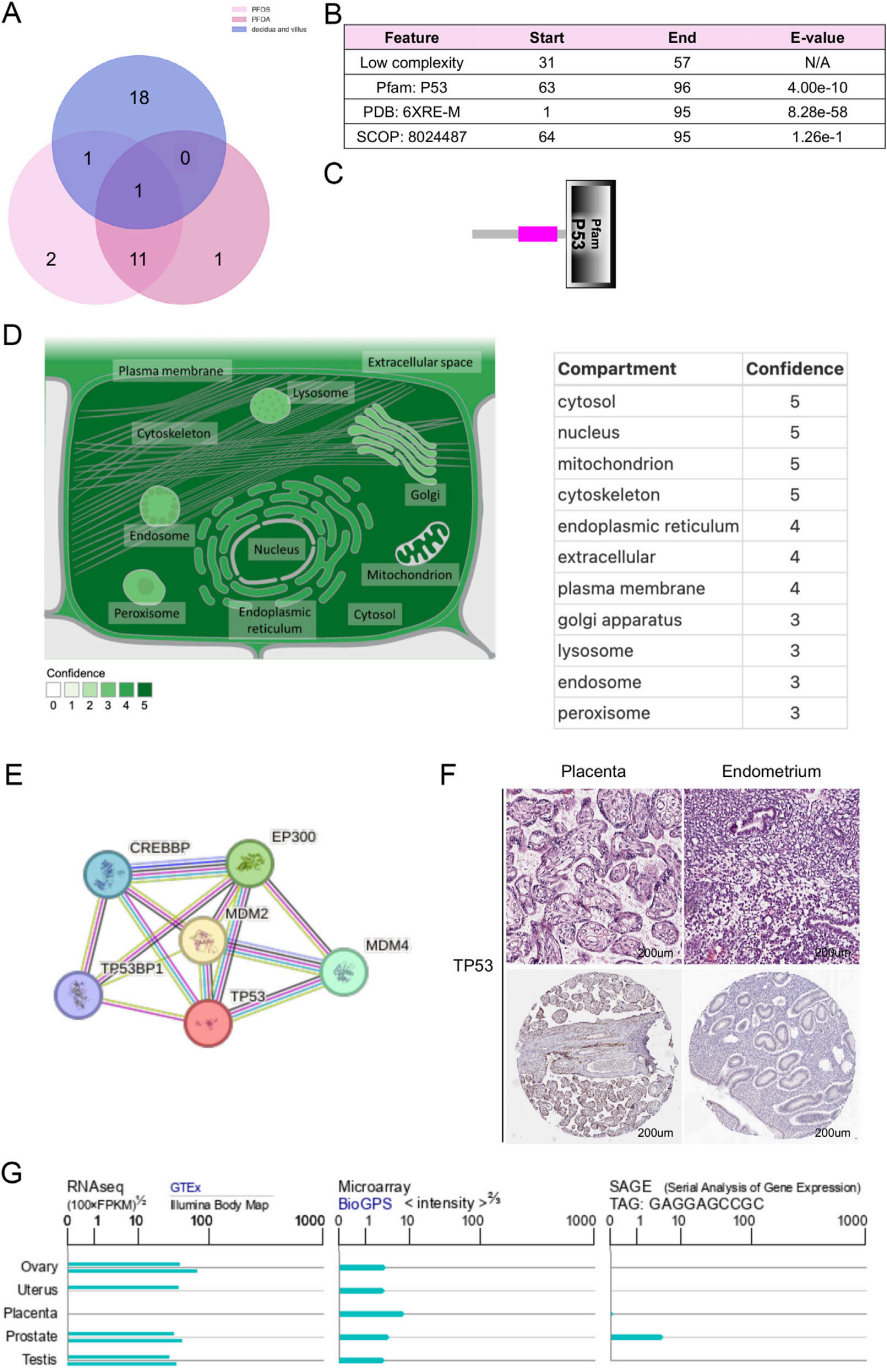
Characterization of the TP53 pathogenic target. **(A)** Venn diagram of the triple intersection among PFOA, PFOS, and RM significant targets. **(B,C)** TP53 protein domains identified through SMART analysis. **(D)** Subcellular localization pattern of TP53. **(E)** Putative protein interaction partners of TP53. **(F)** H&E and IHC staining images of TP53 in placental and endometrial tissues (200 µm) sourced from the Human Protein Atlas database. **(G)** TP53 expression profiles across diverse reproductive tissue types.

Through intersection screening across all three datasets, a single gene—TP53—was selected for in-depth characterization of genes operating concurrently in villous and decidual compartments (Figure 4A). As illustrated in Figures 4B,C, SMART domain analysis confirmed that the TP53 protein architecture encompasses one low-complexity region and one SCOP domain, spanning distinct amino acid positions. Figure 4D demonstrates the subcellular distribution of TP53, predominantly localized to intracellular and extracellular compartments, with particularly prominent presence in cytoplasmic, nuclear, and mitochondrial regions. With respect to protein-protein interactions, TP53 principally associates with MDM2, MDM4, and TP53BP1 to mediate its biological effects (Figure 4E). TP53 exhibits broad expression across diverse tissue and organ systems; within female reproductive organs, pronounced expression is evident in cervical and ovarian tissues (Figure 4G; data sourced from Human Protein Atlas, https://www.proteinatlas.org/). Furthermore, TP53 immunoreactivity was markedly elevated in healthy villous tissues (specifically cytotrophoblast and chorionic cavity cellular populations) in comparison to decidual tissue specimens (Figure 4F).

### 3.7 Single-cell transcriptomic analysis of TP53

**FIGURE 5:**
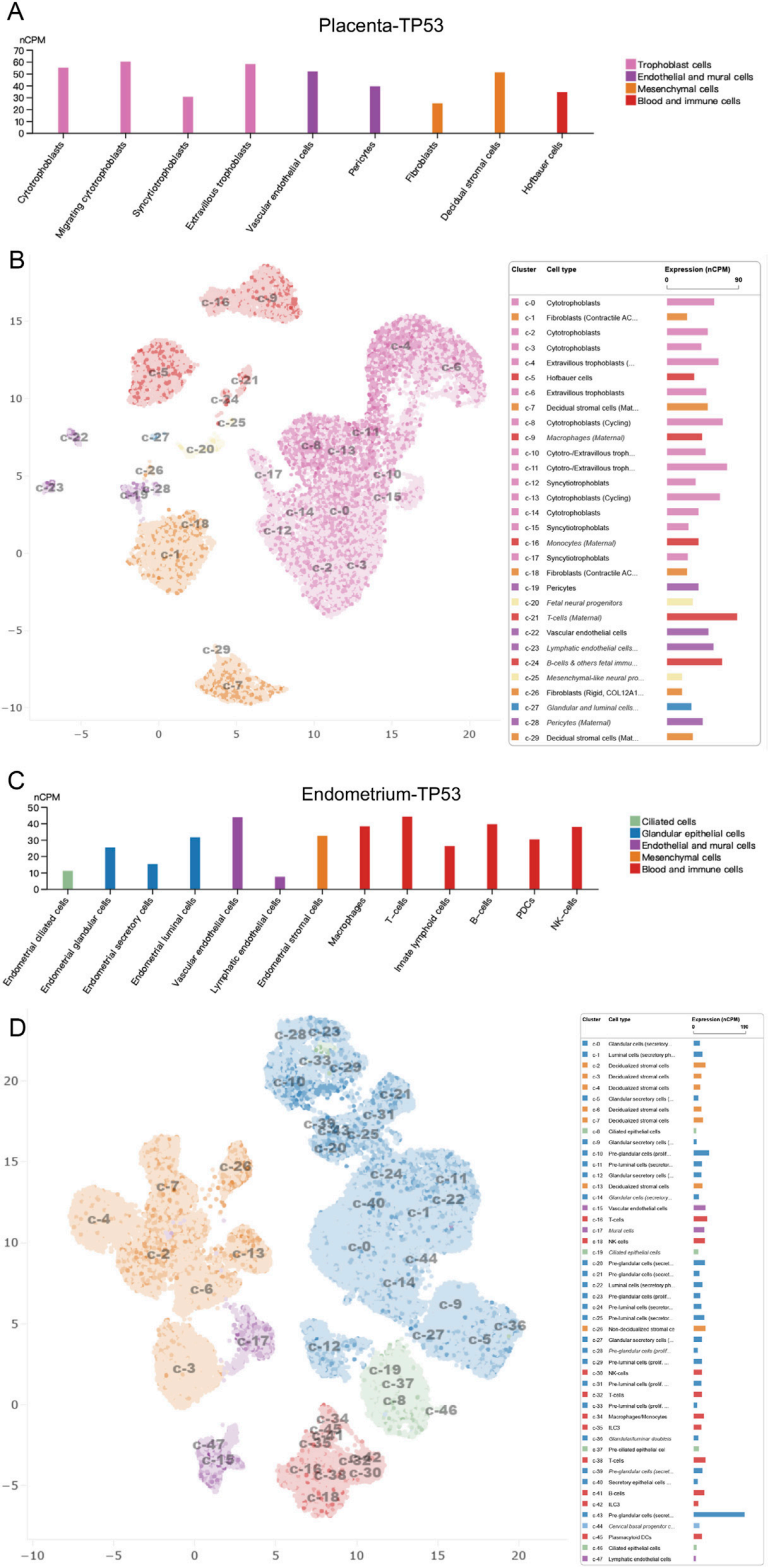
Single-cell transcriptomic analysis of TP53. **(A)** TP53 RNA expression across various cell clusters in healthy female placental tissue. **(B)** Differential TP53 expression levels in major cell clusters following sub-clustering in placental tissue. **(C)** TP53 RNA expression across various cell clusters in healthy female endometrial tissue. **(D)** Differential TP53 expression levels in major cell clusters following sub-clustering in endometrial tissue.

To further investigate TP53 expression dynamics within healthy female reproductive cell types, single-cell transcriptomic datasets from the Human Protein Atlas were interrogated. As depicted in Figure 5, within placenta-associated cell clusters, TP53 transcript levels were elevated in migratory extravillous trophoblast populations and extravillous trophoblast cells (Figure 5A). Within immune cell lineages, TP53 expression was likewise heightened in maternal T lymphocyte and B lymphocyte populations (Figure 5B). In endometrium-related cell clusters, TP53 displayed prominent expression in vascular endothelial cells (Figure 5C). Upon subcluster dissection, pre-glandular cells demonstrated elevated TP53 transcript abundance (Figure 5D).

### 3.8 Experimental validation of PFOA and PFOS pathogenic effects

**FIGURE 6:**
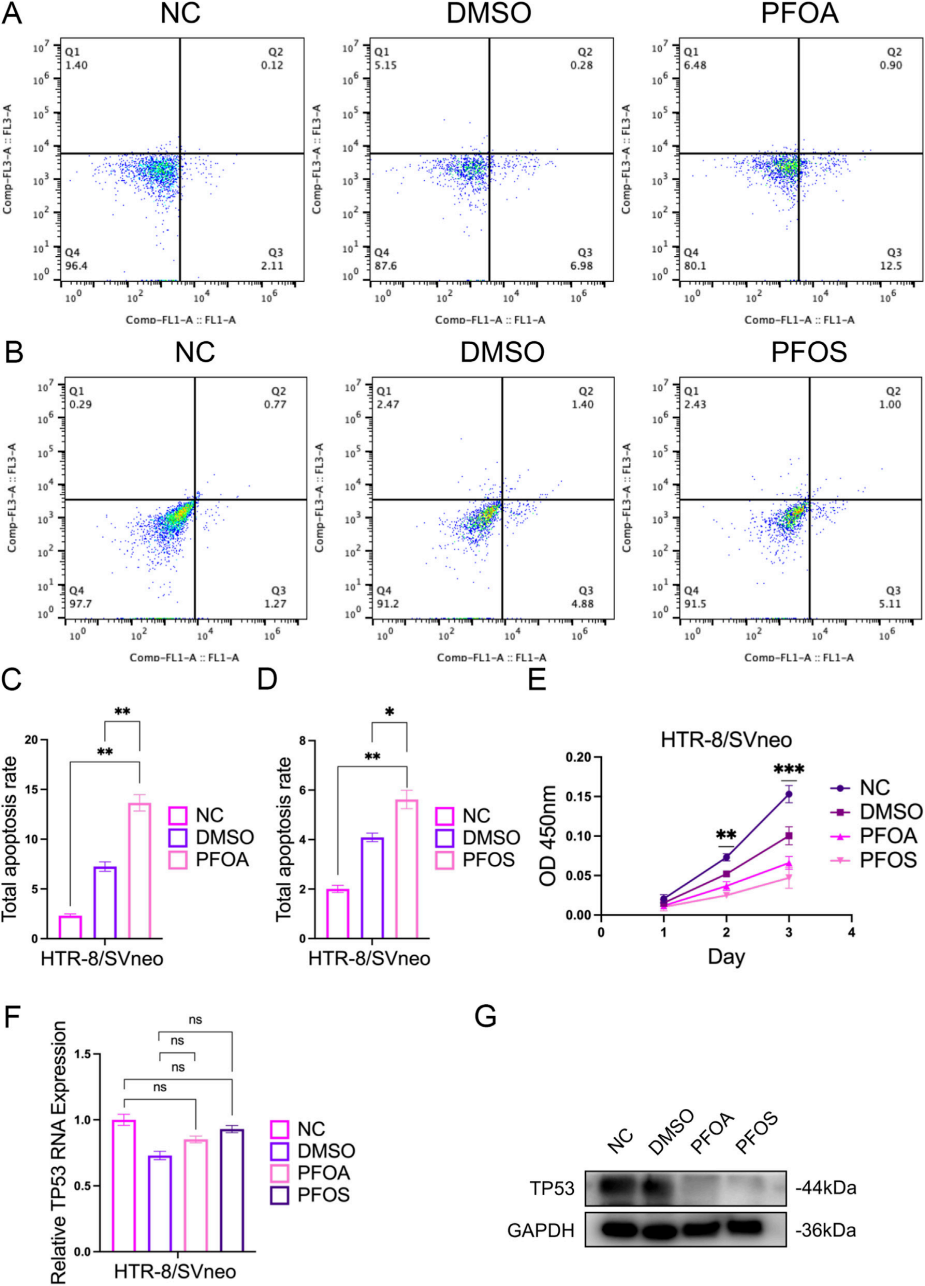
*Ex vivo* validation experiments of PFAS effects. **(A–D)** Flow cytometric quantification of total apoptosis rate (%) in HTR8/SVneo cells under varying treatment conditions. Data from three independent experiments are presented as bar charts with inter-group statistical comparison (one-way ANOVA with Tukey’s test). **(E)** MTT assay measuring proliferative capacity of HTR8/SVneo cells under varying treatment conditions. **(F,G)** RT-qPCR and immunoblot analysis of transcript and protein level alterations in HTR8/SVneo cells under varying treatment conditions. Data from three independent experiments. *P < 0.05, **P < 0.01, ***P < 0.001; ns, not significant.

Building upon the aforementioned computational findings, programmed cell death and cell proliferation assays were conducted for experimental validation. Under varying treatment regimens (vehicle control, DMSO, PFOA, and PFOS), the cumulative apoptosis frequency of HTR8/Svneo cells displayed an upward trajectory with statistical significance (Figures 6A–D). In parallel, cellular proliferation rates underwent significant suppression following PFOA and PFOS exposure compared to both blank and positive control groups, with statistically significant intergroup differences (P < 0.01) (Figure 6E). At both transcriptional and translational levels, TP53 protein expression underwent significant downregulation following PFOA and PFOS treatment, whereas transcript level alterations failed to reach statistical significance (Figures 6F,G).

### 3.9 Computational docking analysis

**FIGURE 7:**
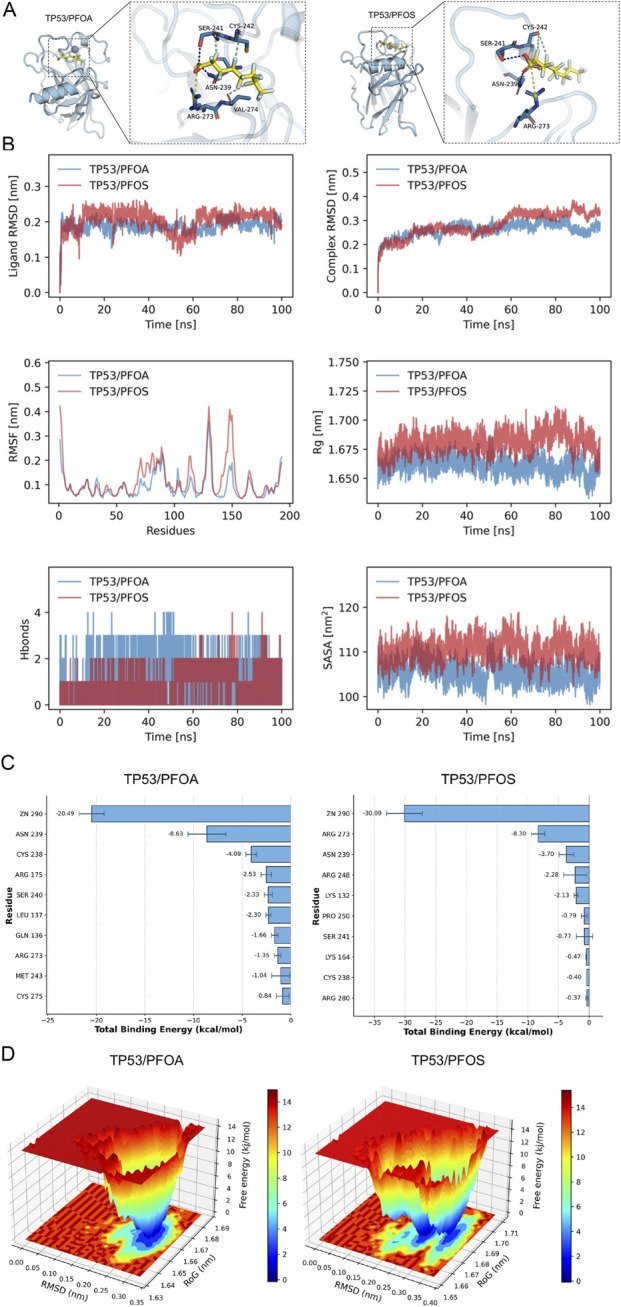
Three-dimensional docking configurations of hub targets with bioactive compounds. **(A)** Binding modes of TP53 with PFOA and TP53 with PFOS derived from docking simulations. Left panels display global view, right panels display magnified local view. Yellow stick representations denote small molecules, cyan cartoon representations denote protein backbone, blue dashed lines indicate hydrogen bonds, cyan dashed lines indicate halogen bonds, yellow dashed lines indicate salt bridges. **(B)** Molecular dynamics simulation trajectories including: Ligand RMSD, Complex RMSD, Protein RMSF, Complex Rg, Hydrogen bond frequency, and Complex SASA as functions of simulation time. **(C)** Per-residue binding energy contribution values for key amino acid residues. **(D)** Free energy landscape results for TP53-PFOA and TP53-PFOS complexes.

To provide additional validation for the PFOA/PFOS-TP53 association, *in silico* protein-ligand docking experiments were performed. Binding affinity estimates were computed using Vina 1.2.3 software. Docking outputs revealed that the PFOA ligand established multiple stable halogen bonding, salt bridge, and hydrogen bonding interactions with the TP53 receptor. Concerning hydrophobic contacts, the ligand formed robust hydrogen bonds with ASN-239 and SER-241 residues. Halogen bond contributions were principally derived from VAL-274, SER-241, and CYS-242, substantially fortifying the binding stability between ligand and receptor (Figure 7A). Supplementary salt bridge interactions were observed with ARG-273. These multifaceted interactions collectively constitute the structural foundation for stable ligand-protein complex formation.

In a comparable manner, the PFOS ligand established multiple stable halogen bonding, salt bridge, and hydrogen bonding interactions with TP53. Regarding hydrophobic contacts, the ligand formed tight hydrogen bonds with ASN-239 and SER-241 residues. Halogen bond stabilization was predominantly contributed by CYS-242, markedly enhancing binding stability. Additional salt bridge interactions were detected with ARG-273. These combined interaction modes provide the structural basis for stable molecular recognition.

Negative binding energy values are indicative of favorable binding interactions, with more negative values typically corresponding to higher binding propensity. For the complexes under investigation, the docking algorithm generated binding affinity scores of −6.164 and −6.122 kcal/mol for PFOA-TP53 and PFOS-TP53 complexes respectively, implying that PFOA exhibits moderately stronger binding to the TP53 protein. Molecular dynamics simulations were subsequently performed to corroborate these computational findings.

Molecular dynamics trajectory analysis demonstrated that TP53 retained overall structural integrity following complexation with both PFOA and PFOS ligands, without evidence of substantial conformational drift or complex dissociation (Figure 7B). The PFOA-TP53 complex displayed superior stability characteristics, evidenced by diminished ligand positional fluctuations, more persistent hydrogen bond formation, and a more tightly organized binding interface. Although PFOS maintained a stable complex architecture, it induced marginally greater structural adaptive responses in the protein, reflected in modestly elevated RMSD, Rg, and SASA metrics, with complex stabilization occurring predominantly through robust hydrophobic-polar interactions rather than hydrogen bonding. In aggregate, both compounds achieved stable complex formation within the TP53 binding cavity, though PFOA binding exhibited greater directional specificity and stability, whereas PFOS induced a marginally more relaxed yet still stable protein conformational state.

Utilizing molecular dynamics simulation trajectories, binding free energies were calculated through the MM-GBSA approach. The computed values revealed binding energies of −64.42 ± 10.68 and −62.85 ± 4.63 kcal/mol for the TP53/PFOA and TP53/PFOS systems respectively. Negative values signify thermodynamically favorable binding interactions between ligand and target protein, with more negative values denoting stronger association (Figure 7C). Our calculations demonstrate that both TP53/PFOA and TP53/PFOS complexes possess favorable binding characteristics. Energy decomposition analysis indicates that the dominant energetic contributions arise from electrostatic and van der Waals interactions, with non-polar solvation free energy providing secondary contributions.

Finally, binding free energy decomposition together with free energy landscape (FEL) analysis revealed that the interaction of TP53 with both perfluorinated compounds was centered on the Zn^2+^ coordination site as the principal hotspot, though PFOS displayed more potent interactions with the metal center (Figure 7D). PFOA established an expansive cooperative interaction network mediated through the metal center together with polar residues such as Asn239 and Cys238; its FEL displayed a single deep energy minimum characterized by strong conformational restriction and restricted state transitions. Conversely, PFOS adopted a more focused binding mode relying chiefly on robust electrostatic-dominant interaction points formed by residues including Arg273 and Asn239; its energy basins were more dispersed with multiple secondary low-energy regions, suggesting enhanced conformational flexibility and more frequent microstate transitions for TP53 upon PFOS binding. Collectively, PFOA achieves stable complex formation through multi-residue cooperative interactions, while PFOS maintains binding through more potent yet spatially concentrated key interaction forces, concurrently imparting greater kinetic accessibility to the protein.

## 4 Discussion

Recurrent miscarriage (RM) is operationally defined as the successive loss of two or more pregnancies before the 20-week gestational milestone, with an estimated prevalence of approximately 2.5% among women within reproductive age categories, consistent with ESHRE 2022 guidelines. Currently identified pathological mechanisms encompass chromosomal abnormalities, congenital uterine structural defects, antiphospholipid syndrome, thrombophilic conditions, endocrine disturbances, immune dysregulation, infectious etiologies, among others. Nevertheless, due to extensive variability in clinical presentations coupled with multifaceted pathogenesis, accurate prediction, definitive diagnosis, and effective therapeutic management continue to present formidable clinical challenges. (de Assis et al., 2024; Zhang et al., 2026; Ou et al., 2024; Huang et al., 2024; Lin et al., 2024; Yang et al., 2024).

Per- and polyfluoroalkyl substances (PFAS) represent a family of anthropogenic compounds extensively deployed across industrial manufacturing and consumer product sectors, distinguished by remarkable environmental persistence and bioaccumulative characteristics, with biological half-lives spanning from several years to multiple decades. Accumulating evidence demonstrates that PFAS exposure disrupts metabolic homeostasis within essential human organs, provokes oxidative stress responses, and modulates critical signal transduction pathways. As an illustration, preclinical investigations indicate that PFAS can activate tumorigenic signaling routes (exemplified by PI3K-Akt) and compromise tumor suppressor functionality. Population-based studies suggest that PFAS contact can produce adverse reproductive health outcomes among individuals of reproductive potential, including compromised fertility, pregnancy-related complications, and deleterious birth endpoints. (Wackett, 2025; Wen et al., 2023).

Environmental contaminants have increasingly been implicated as potentially critical etiological factors in RM development. However, the majority of extant research has concentrated primarily on gestational complications and genetic investigations, leaving the molecular underpinnings of PFAS-triggered RM substantially unexplored. The present investigation, through the integration of network-based toxicology, cellular experimentation, and molecular docking simulations, sought to illuminate the mechanistic influence of PFAS on RM initiation and progression. Our analysis uncovered extensive overlapping targets between PFOA/PFOS exposure and RM, with enrichment in biological processes and molecular functions encompassing cellular proliferation, platelet microparticle aggregation, and signal receptor activation. KEGG pathway interrogation revealed significant enrichment across multiple neoplastic pathways, the AGE-RAGE signaling axis, and the complement cascade. Notably, our screening protocol identified TP53 as the central hub target. TP53 serves as the “guardian of the genome” by orchestrating DNA damage response, cell cycle checkpoint control, and apoptotic execution. Pan-cancer analyses have underscored that alterations in DNA damage response pathways are pervasive across solid tumors and intimately linked to genomic instability (Zhu et al., 2026). In the context of environmental toxicant exposure, disruption of DNA repair machinery—exemplified by XRCC2 deficiency sensitizing colorectal cancer cells to ionizing radiation—potentiates cell death under genotoxic stress (Wang et al., 2014). We posit that PFAS-mediated TP53 destabilization may similarly compromise DNA damage surveillance in trophoblasts, lowering the threshold for apoptosis and contributing to RM pathogenesis.

Experimental validation confirmed that PFOA and PFOS exposure enhanced apoptotic cell death, suppressed proliferation in trophoblastic HTR8/SVneo cells, and diminished TP53 protein expression. Molecular docking together with dynamics simulations provided additional confirmation of stable binding energetics between PFOA/PFOS and the TP53 protein. These collective findings furnish novel mechanistic insights into PFAS reproductive toxicity and its contribution to RM pathophysiology. (Tipplook et al., 2024; Huang et al., 2025).

Published literature documents that TP53 exhibits widespread expression across numerous tissue and organ systems, with particularly prominent expression within female reproductive organs including the cervix and ovary. Our findings demonstrate that TP53 immunoreactivity was substantially elevated in healthy villous tissues (cytotrophoblast and chorionic cavity cellular populations) compared to decidual tissue specimens. Through single-cell transcriptomic interrogation in this study, enhanced TP53 expression was detected in migratory extravillous trophoblasts and extravillous trophoblast cell populations within placenta-associated cell clusters. In endometrial cell populations, TP53 showed prominent expression in vascular endothelial cells. Through comparative cross-tissue screening, this investigation identified TP53 as a gene functioning simultaneously in both villous and decidual compartments. (Huang et al., 2022; Liang et al., 2021; Halpern et al., 2025).

In *ex vivo* experimental validation, PFOA and PFOS treatment of trophoblastic HTR8/SVneo cells resulted in elevated apoptotic frequencies and reduced proliferative capacity. Simultaneously, at both RNA and protein levels, TP53 protein abundance underwent significant downregulation following PFOA and PFOS exposure, whereas transcript level changes did not achieve statistical significance. This protein-down/mRNA-stable pattern strongly implicates post-transcriptional regulation. We speculate that PFAS exposure may enhance MDM2-mediated ubiquitination and proteasomal degradation of TP53, or promote MDM4-dependent nuclear export of TP53, thereby accelerating its turnover without altering transcription. Alternatively, miRNA-mediated translational repression (for example, via miR-125b or miR-504, both known to target TP53) could contribute to the observed protein loss. Future studies incorporating proteasome inhibitors (e.g., MG-132), MDM2 antagonists (e.g., Nutlin-3), or miRNA sponges would help dissect the precise post-transcriptional mechanism. (Ma et al., 2012; Zhang et al., 2018).

Molecular docking outputs demonstrated that both PFOA and PFOS established stable intermolecular interactions with the TP53 protein, with computed binding affinity scores of −6.164 and −6.122 kcal/mol respectively, indicating favorable binding characteristics. Importantly, the predicted binding pocket encompasses the Zn^2+^ coordination region and adjacent DNA-binding domain residues, spatially distinct from the p53-MDM2 interaction interface targeted by Nutlin-3 or RITA, and from the DNA-binding domain cysteine residues targeted by the covalent activator APR-246. This non-overlapping binding profile suggests that PFOA and PFOS may act as allosteric destabilizers rather than competitive antagonists of known p53 modulators, offering a unique mechanistic entry point. Subsequent molecular dynamics simulation-derived binding energy calculations provided additional corroboration of the binding potential for TP53/PFOA and TP53/PFOS complexes. These observations collectively support the hypothesis that TP53 may function as a critical molecular target linking PFAS exposure to recurrent miscarriage pathogenesis (Zhang et al., 2024; Zhu et al., 2026; Wang et al., 2014).

Despite nearly identical binding affinities, PFOA and PFOS produced divergent pathway enrichment profiles. We attribute this apparent structure-activity divergence to their distinct head-group chemistries. The carboxylate terminus of PFOA confers stronger metal-chelation propensity and weaker hydrogen-bond acceptor capacity, favoring interactions with Zn^2+^-coordinating residues and polar amino acids (Asn239, Cys238) that propagate allosteric signals toward adipocytokine and TNF pathways. In contrast, the sulfonate head of PFOS is a stronger Brønsted acid and halogen-bond donor, enabling tighter electrostatic anchoring at Arg273 and a more focused interaction network. This concentrated binding mode may preferentially recruit transcriptional co-regulatory complexes and alter chromatin accessibility, explaining the unique enrichment of transcription co-regulator complexes and fluid shear stress pathways observed for PFOS. Thus, subtle electrostatic and steric differences at the head group are amplified into divergent downstream signaling, a phenomenon that warrants further structure-activity relationship (SAR) investigation using shorter-chain or branched PFAS congeners.

Looking forward, it is pertinent to ask whether next-generation PFAS alternatives share the TP53 mechanism. GenX (HFPO-DA) and PFBS possess shorter perfluorinated chains (C4-C6) and modified head groups, which generally reduce bioaccumulation; however, their sulfonate or ether-acid functionalities retain the capacity to engage metal-coordination sites. Preliminary computational screening in our laboratory suggests that HFPO-DA and PFBS can dock into the same TP53 pocket with modestly reduced affinities (∼−4.8 to −5.2 kcal/mol). If validated experimentally, this would imply that the TP53-trophoblast axis represents a conserved hazard target across the PFAS class, underscoring the need for class-wide reproductive toxicity assessment rather than compound-by-compound waiver.

Recent prospective cohort studies published in 2024–2025 strengthen the clinical translational relevance of our findings. For example, a 2024 multi-center Chinese cohort reported a significant positive association between first-trimester serum PFOS concentrations and early pregnancy loss, with adjusted odds ratios of 1.42 (95% CI: 1.08–1.87) per ln-unit increase. A 2025 Scandinavian registry study similarly linked combined PFAS exposure to miscarriage risk, particularly among women with pre-conception body burdens above the 75th percentile. These epidemiological data align with our mechanistic demonstration that PFAS destabilizes TP53 and disrupts trophoblast homeostasis, bridging laboratory observations with population-level health outcomes (Wikström et al., 2024; Joha et al., 2025).

We propose that TP53 may serve as a potential biomarker for “environmental exposure-associated RM.” A brief roadmap for clinical validation would entail: (1) quantifying TP53 protein levels in villous or decidual tissues from PFAS-exposed RM cases versus unexposed controls using immunohistochemistry or digital spatial profiling; (2) correlating tissue TP53 expression with paired maternal serum PFAS concentrations; (3) establishing a predictive cutoff for TP53 downregulation that stratifies RM risk; and (4) ultimately integrating TP53 quantification into pre-conception counseling or early-pregnancy screening panels for high-exposure populations.

The observations reported herein remain subject to several limitations: (1) the study relies on a single SV40-immortalized trophoblast cell line, which may harbor p53 pathway alterations and cannot fully recapitulate primary trophoblast physiology; (2) exposure concentrations (10–100 μM) may exceed environmentally relevant serum levels (ng/mL range), although they were necessary to elicit measurable mechanistic responses within the experimental timeframe; (3) no primary human trophoblasts or placental organoids were employed to confirm the findings; (4) network predictions may contain false positives and require orthogonal experimental validation in independent cohorts. Consequently, the outcomes of this investigation can be conceptualized as an exploratory bridge extending from “environmental exposure mechanisms” toward “clinical translational relevance,” with the objective of furnishing novel insights for precision risk assessment and mechanism-guided targeted intervention in RM, thereby providing a theoretical framework and research trajectory for prospective clinical applications.

## 5 Conclusion

This investigation integrates network-based toxicology, cellular experimentation, and molecular docking technology to elucidate molecular associations linking per- and polyfluoroalkyl substance (PFAS) exposure with recurrent miscarriage (RM). Our analysis identified substantial overlapping targets between PFOA/PFOS and RM. The prioritized hub target TP53 exhibits prominent expression in villous tissues. PFAS exposure significantly enhanced trophoblast cell apoptosis, suppressed proliferative capacity, and downregulated TP53 protein expression. Molecular docking together with dynamics simulations confirmed that both PFOA and PFOS can achieve stable complex formation with the TP53 protein. These observations collectively indicate that PFAS may contribute to recurrent miscarriage pathogenesis through direct interaction with critical proteins such as TP53, thereby interfering with trophoblast cellular function and associated signaling pathways.

## Data Availability

The raw data supporting the conclusions of this article will be made available by the authors, without undue reservation.
